# Cricopharyngeal Myotomy; a Rescue Surgery for Dysphagia in Lateral Medullary Syndrome

**DOI:** 10.1007/s12070-022-03165-3

**Published:** 2022-11-06

**Authors:** Vijendra Shenoy S, Kshithi K, Navya Parvathareddy, Saksham Dhawan

**Affiliations:** Dept of ENT and Head & neck surgery, Kasturba Medical College, Manipal Academy of Higher Education, Mangalore, India

**Keywords:** Lateral medullary syndrome, dysphagia, cricopharyngeal myotomy, esophagus, infarction

## Abstract

Lateral medullary syndrome/Wallenberg syndrome, is a neurological disorder occurring due to ischemia in the lateral part of medullary oblongata resulting in wide range of symptoms. Dysphagia is usually exhibited in severe and persistent form in LMS. Hence timely intervention is mandatory before the patient further worsens. We describe a case of Lateral medullary syndrome with persisitent dysphagia who was managed successfully with cricopharyngeal myotomy.

## Introduction

Lateral medullary syndrome (LMS) also known as Wallenberg syndrome, is a clinical syndrome caused by an acute ischemic infarct of the lateral medulla oblongata. This infarct is usually attributed by crossed hemisensory disturbance, ipsilateral Horner syndrome, and cerebellar signs [1]. Most common complaint in a LMS patient is dysphagia, which in turn leads to aspiration pneumonia, malnutrition, increased mortality and prolonged hospital stay [2, 4]. Hence timely intervention is mandatory before the patient further worsens. Pathophysiology is unclear, it is said that the medullary central pattern generators coordinate the pharyngeal phases of swallowing which are in turn controlled via neural connections. These connections are altered in LMS. Approach to and prognosis of a patient with dysphagia in LMS varies from patient to patient. Best approach to such cases is still debatable. Here we report a case of LMS with dysphagia treated with surgical management, which resulted in improvement in swallowing function and quality of life.

## Case Report

A 46 years old male with no known comorbidities presented to neurologist with acute onset of difficulty in swallowing associated with severe cough suggesting aspiration, left sided weakness, giddiness. Upon further examination and necessary investigations, the patient was diagnosed to have lateral medullary syndrome. Patient was started on IV antibiotics and enteric nutrition was continued with nasogastric tube feeding. Patient was given swallowing rehabilitation under supervision of a swallow therapist. Patient persisted to have dysphagia, more for liquid and was also associated with severe cough even after 6 weeks of rehabilitation. Reference to ENT was given in view of persistent dysphagia.

**Image 1:**
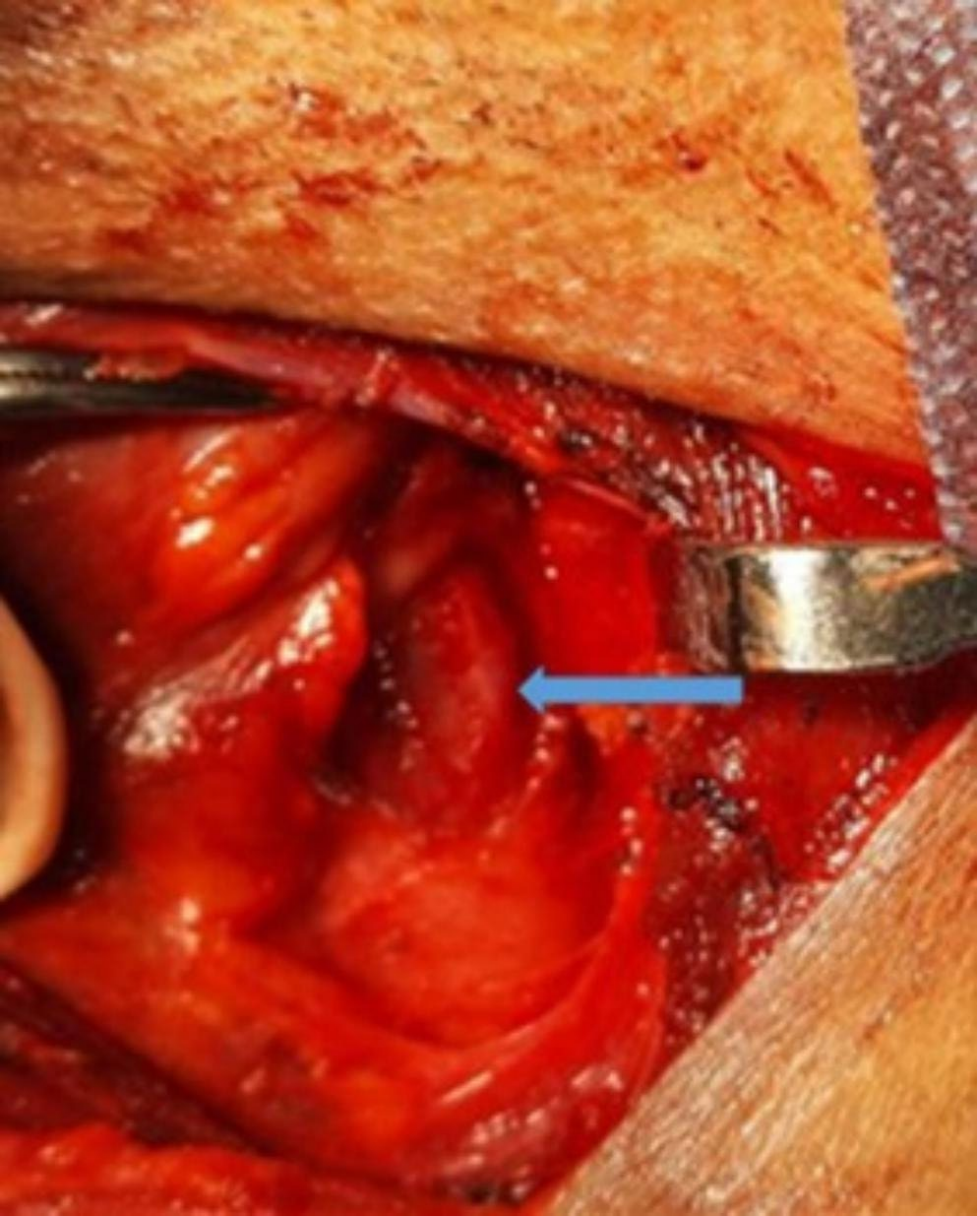
Showing incision over the cricopharyngeus muscle until visualization of markings over the ET tube

Patient underwent a thorough dysphagia assessment. Anderson dysphagia score was 35. Functional endoscopic evaluation of swallowing was done, which showed pooling of food bolus despite multiple swallowing efforts. Manometric assessment was suggestive of cricophayngeal sphincter spasm. Fluoroscopic evaluation ruled out aspiration. Further patient was planned for cricopharyngeal myotomy. After pre-operative fitness was obtained and written informed consent was taken. Following nasal intubation, prior to definitive surgery patient underwent esophagoscopy to rule out tumors or strictures of the distal sites. A horizontal given at the level of cricoid cartilage given. Dissection was carried out along anterior border of SCM till prevertebral fascia was reached, larynx and esophagus were exposed, cricothyroid joint palpated, laryngotracheal complex rotated by placing a double pronged hook. Anaesthesia team was requested for midway esophageal intubation for better palpation and identification of esophagus during surgery. The cricopharyngeus muscle was easily palpated and visualized stretched over the endo tracheal tube (ET tube). Cricopharygeus muscle cut until the underlying oesophageal mucosa came into view **(Image **1**)**. Visualization of markings over the ET tube denoted adequate thinning of cricopharyngeal muscle. Hemostasis ensured and drain placed and wound closed in layers. Ryle’s tube feeds were started on the same day. Drain was removed after 3 days and improvement noted gradually. By the end of 1st week the patient got symptomatically better. Ryle’s tube feed was continued for 2 weeks. At the end of 2 week, he was given trial feeds and was able to swallow with effort. Ryle’s tube was removed. He underwent post operative manometric assessment which showed relief of sphincter spasm and FEES showing improved swallowing function. At 2 weeks Anderson score was 60 and at 6 weeks it was 95. After 6months of follow up, a significant weight gain was noted in the patient.

## Discussion

Lateral medullary syndrome/Wallenberg syndrome, is a neurological disorder occurring due to ischemia in the lateral part of medullary oblongata resulting in wide range of symptoms. Most commonly vertebral artery/ PICA gets blocked leading to vertigo, dizziness, nystagmus, ataxia, nausea, vomiting, dysphagia and hiccups [3]. Dysphagia is usually exhibited in severe and persistent form in LMS [6] and so far not much studies are noted on dysphagia in brain stroke patients. Hence timely intervention is mandatory before the patient further worsens. Approach to and prognosis of a patient with dysphagia in LMS varies from patient to patient. In a recent study it has been stated that 40–47% of patients developed dysphagia as a complication of brain stem stroke [6].

Most common ENT complaint in a patient with LMS is dysphagia, other complaints include dysphonia, facial pain, headache, vertigo, dysarthria, nystagmus. Treatment for dysphagia according to recent study [6, 7] involves traditional therapy (diet modification, exercises to strengthen the oropharyngeal musculature, compensatory maneuvers to facilitate laryngeal elevation and closure during swallowing, and techniques to stimulate and strengthen the swallowing reflex) and advanced surgical procedures i.e. cricopharyngeal myotomy [5]. The myotomy is commonly done via an external approach, although an endoscopic approach using CO2 laser can also be employed. Alternative treatments include dilatations and Botulinum toxin injections.

Cricopharyngeal myotomy aims to remove the obstructive effect of the upper esophageal sphincter by nicking the cricopharyngeus muscle. Indications include cricopharyngeal dysfunction, Zenker’s diverticulum, glottic insufficiency, intractable aspiration & sialorrhea.

Although there are no specific criteria to make decisions on whether myotomy is the best approach among all possible procedures. External approach is safer and easier technique to employ. Complications are usually less in this technique. Some of the complications reported in the literature are pharyngeal leak, recurrent laryngeal nerve injury, recurrent dysphagia due to incomplete division, fistula or abscess formation. Post-surgery external approach provides promising results and drastic improvement is noted during follow up, with promising long-term results. This report aims to document the successful outcome obtained post external cricopharyngeal myotomy in a patient with lateral medullary syndrome.

## Conclusion

Approach to dysphagia in a lateral medullary syndrome is often debatable. Early diagnosis and timely intervention has a higher success rate in improvement of patient. External cricopharyngeal myotomy on a LMS patient with dysphagia provides promising results.
